# Post-COVID Myocarditis in Patients with Primary Cardiomyopathies: Diagnosis, Clinical Course and Outcomes

**DOI:** 10.3390/genes15081062

**Published:** 2024-08-12

**Authors:** Olga Blagova, Yulia Lutokhina, Evgeniya Kogan, Polina Savina, Svetlana Aleksandrova, Elena Zaklyazminskaya

**Affiliations:** 1 V.N. Vinogradov Faculty Therapeutic Clinic, I.M. Sechenov First Moscow State Medical University (Sechenov University), 119991 Moscow, Russia; blagovao@mail.ru (O.B.); polina24104@gmail.com (P.S.); 2 Department of Pathology, I.M. Sechenov First Moscow State Medical University (Sechenov University), 119991 Moscow, Russia; koganevg@gmail.com; 3 Department of Radiology, A. N. Bakulev National Medical Research Center for Cardiovascular Surgery of the Russian Ministry of Health, 121552 Moscow, Russia; svaleksandrova@yandex.ru; 4 Laboratory of Medical Genetics, B.V. Petrovsky Russian Research Center of Surgery, 119991 Moscow, Russia; helenezak@gmail.com

**Keywords:** myocarditis, cardiomyopathy, COVID-19, SARS-CoV-2, endomyocardial biopsy, inflammation, arrhythmia, heart failure

## Abstract

The aim of this study was to evaluate the clinical course and outcomes of post-COVID myocarditis in patients with cardiomyopathies (CMP). This case series includes 10 patients with different CMPs who had COVID-19 (seven men; 48.4 ± 11.4 yr.): left ventricular non-compaction (n = 2), arrhythmogenic right ventricular CMP in combination with a heterozygous form of hemochromatosis (n = 1, *HFE*), restrictive CMP (n = 1, *MyBPC3*), laminopathy (n = 1, *LMNA*), dilated cardiomyopathy (n = 1, *MYH7 + MyBPC3*), Danon’s disease (n = 1, *LAMP2*) and AL cardiac amyloidosis (n = 3). Myocardial morphological examination with immunohistochemical staining and PCR for SARS-CoV-2 and cardiotropic viruses was performed in six patients, while cardiac MRI and anti-cardiac antibody titres were evaluated in all patients. Post-COVID lymphocytic myocarditis was confirmed morphologically in six patients (with LVNC, RCM, ARCV, Danon’s disease, and AL amyloidosis). Spike and nucleocapsid coronavirus proteins were detected in cell infiltrates, endothelium and cardiomyocytes in all biopsies; SARS-CoV-2 RNA was found in five out of six. In four patients, the diagnosis of myocarditis was based on MRI, high titres of anti-cardiac antibodies and clinical data. The mean time from COVID-19 to the diagnosis of myocarditis was 7 (5; 10.5) months. Myocarditis manifested with the onset/increase of arrhythmias and heart failure. Immunosuppressive therapy with corticosteroids was administered to six patients and led to an increase in ejection fraction and improvement of heart failure symptoms in five of them. CMPs are a favourable background for the development of post-COVID myocarditis. The onset or deterioration of heart failure and/or arrhythmias in patients with CMPs after COVID-19 requires the exclusion of myocarditis and, if present, the administration of immunosuppressive therapy.

## 1. Introduction

The emergence of a new coronavirus infection (COronaVIrus Disease 2019, COVID-19) has placed patients with primary myocardial disease in a high-risk situation due to the initial severity of their condition, the high incidence of cardiac lesions within COVID-19, and the potentiation of thrombotic complications (already not uncommon in many cardiomyopathies, CMP). A few reports have shown that the incidence of COVID-19 in patients with CMP was not higher than in the general population (1.1% vs. 1.4%), but hospitalisation was more frequent (53.8% vs. 16.5%, *p* = 0.002) and more patients reported a worsening of their health (32.3% vs. 13.2%, *p* = 0.004) [1]. Of 70 patients with hypertrophic cardiomyopathy (HCM) who had COVID-19, 20% were hospitalized and the mortality rate was 2.9% [2]. Similar data are not available for other CMPs.

The development of coronavirus myocarditis on the background of CMP could be expected along with non-specific mechanisms of decompensation. However, proven SARS-CoV-2 (severe acute respiratory syndrome-related coronavirus 2) induced (acute, post-COVID and post-vaccine) myocarditis in patients with CMP is rarely described in the literature. There are only descriptions of probable acute coronavirus myocarditis in a patient with dilated cardiomyopathy due to laminopathy [3] and vaccination-related myocarditis in a patient with arrhythmogenic cardiomyopathy [4]. We would also like to mention our description of a case of ANO5-related HCM with distal myopathy and postvaccination myopericarditis [5].

At the same time, the problem of the combination of dilated CMP (DCM) and myocarditis is at the centre of interest of myocardial disease specialists. Whole-exome sequencing in 12 children with morphologically confirmed myocarditis and DCM phenotype revealed probably pathogenic variants and variants with unspecified clinical significance in genes associated with CMP (titin, troponin, desmocolin, myosin, fibrillin, etc.) in 75% of cases and in immune response genes in 25% of cases [6]. Children with myocarditis and the presence of mutations in CMP genes have been shown to have significantly worse outcomes than those with myocarditis without DCM [7].

The COVID-19 pandemic has highlighted this problem in a new way. It has revealed many peculiarities in the diagnosis and clinical course of myocarditis in the context of CMP and raised the question of its treatment. The absence of such descriptions in the literature may be explained by the poor availability of morphological verification of myocarditis. It is extremely difficult to diagnose superimposed myocarditis in CMP patients without a biopsy because of the similarity in clinical presentation. In this situation, both individual descriptions of confirmed coronavirus myocarditis in CMP and analyses of a series of such cases are highly relevant. Here, we present a case series with separate descriptions of the most representative cases.

**Purpose:** To evaluate the clinical manifestations, diagnostic features and outcomes of post-COVID myocarditis in patients with CMPs

## 2. Materials and Methods

This work was carried out in the V.N. Vinogradov Faculty Therapeutic Clinic. The study included 10 patients with CMP who had COVID-19 (7 men, mean age 48.4 ± 11.4, from 22 to 72 years). The mean follow-up was 6 [6.0; 9.5] months.

The spectrum of CMPs included a heterozygous form of haemochromatosis combined with probable arrhythmogenic CMP (n = 1), left ventricular non-compaction (LVNC, n = 2), Danon’s disease (n = 1), laminopathy (n = 1), dilated CMP (n = 1), restrictive CMP (n = 1), and AL-amyloidosis (n = 3). A family history of cardiomyopathy was noted in 6 patients, including confirmed Danon’s disease in the patient’s son (who died of end-stage heart failure) and DCM in the patient’s mother (who successfully underwent heart transplantation).

The diagnosis of COVID-19 was confirmed by a positive result of a nasopharyngeal swab for SARS-CoV-2 by PCR in 6 patients (the other 4 were not tested or were negative) and a subsequent increase in IgG to SARS-CoV-2 in all patients. In addition to COVID-19, the development of myocarditis in 2 patients was preceded by vaccination with Gam-COVID-Vac (4 months before and after disease onset, respectively). In one patient with LVNC and myocarditis, peripartum CMP could not be excluded (decompensated one month after delivery). Apart from her, all patients had COVID-19 before the beginning of 2022. The disease was severe in one patient (treated at home and no CT was performed), and two were hospitalized due to uncontrolled fever. In three patients, CT was performed (all had CT1). The mean time from COVID-19 disease to diagnosis of myocarditis was 11 [5.5; 19.5] months.

All patients underwent electrocardiography (ECG), echocardiography (EchoCG), Holter monitoring (HM), magnetic resonance imaging (MRI) of the heart in 6 patients, and the level of anti-cardiac antibodies (antibodies to antigens of cardiomyocyte nuclei (ANF) and antibodies to antigens of endothelium, cardiomyocytes, smooth muscle, and fibres of the conduction system) in the blood was assessed by indirect immunofluorescence analysis in all patients. Hemodynamically significant coronary atherosclerosis was ruled out in all patients over 40 years of age.

The diagnosis of post-COVID myocarditis was based on data from myocardial morphological examination (5 endomyocardial biopsies (EMBs) and 1 intraoperative biopsy were performed) and/or cardiac MRI in combination with medical history, echocardiography and elevated anti-cardiac antibodies titres. Morphological examination of the myocardium included haematoxylin and eosin, Van Gieson, Perls, Congo red and PAS staining; immunohistochemistry (IHC) with antibodies against CD3, CD20, CD45, CD68, SARS-CoV-2 nucleocapsid and spike proteins; and PCR for parvovirus B19 DNA, herpesviruses and SARS-CoV-2 RNA (QuantiTect single-step PCR kit (Qiagen, Hilden, Germany) and Primer-BLAST program).

DNA diagnostics. DNA was extracted from venous blood according to standard protocols. For 4 patients, whole exome sequencing (WES) was performed using the Illumina platform (NextSeq 550Dx or NovaSeq 6000, Illumina, San Diego, CA, USA) with a SureSelect Human All Exon V7 (Agilent Technologies, Santa Clara, CA, USA) enrichment kit based on the manufacturer’s instruction. Reads were aligned to the human genome build GRCh37/UCSC hg19 and variant calling used an automatic module EMSEMPLE_VEP with the following analysis of the sequence variants using a custom-developed bioinformatics pipeline.

Confirmation of genetic findings in probands and cascade familial screening was performed by capillary Sanger resequencing on an ABI 3500 DNA Analyzer (Thermo Fisher Scientific, Waltham, MA, USA) according to the manufacturer’s instructions.

For 2 patients with suspected syndromic diagnosis (Danon’s disease and hemochromatosis) capillary Sanger sequencing of the coding areas of the target genes (*LAMP2* and *HFE*) was performed with the same instrument.

Pathogenicity assessment of all variants confirmed by Sanger sequencing was performed according to ACMG (2015) criteria (on behalf of the ACMG Laboratory Quality Assurance Committee) [8].

Statistical analysis was performed using IBM SPSS statistics v.22. Discrete data are presented as absolute value and percentages, continuous data are presented as arithmetic mean ± standard deviation in cases of a normal distribution or as quartiles 50 (25; 75).

Ethical Committee. All patients signed informed consent forms for investigations (including myocardial biopsy and various types of immunosuppressive therapy) approved by the local ethical committee of Sechenov University (Protocol 10-22, 19.05.22) in accordance with the guidelines in the Declaration of Helsinki.

## 3. Results

**Figure 1:**
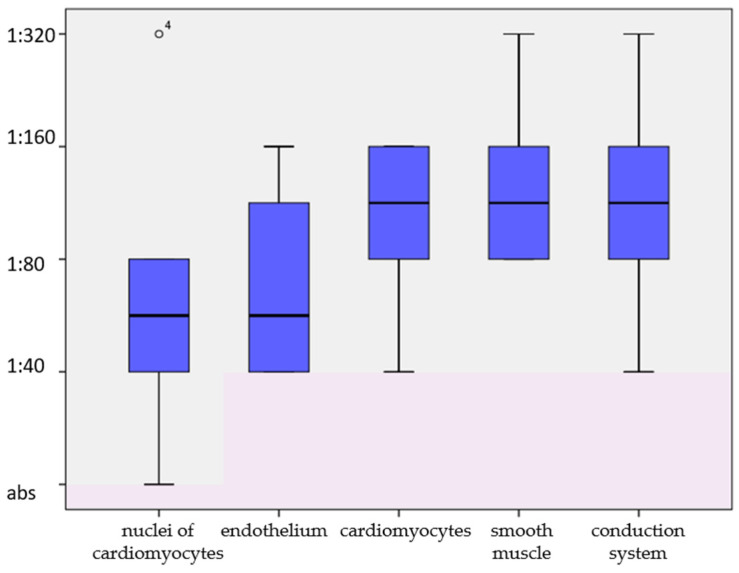
The level of the anti-cardiac antibodies in patients with cardiomyopathies and post-COVID myocarditis.

The development of post-COVID myocarditis was suspected by the occurrence/progression of chronic heart failure (CHF) in five/five patients, premature ventricular contractions (PVCs) in four patients, atrial fibrillation (AF) in four patients and atrial flutter in one patient. No association was observed between the severity of COVID-19 and subsequent myocarditis. Anti-cardiac antibody titres were elevated three to four-fold in all patients except the patient with amyloidosis, who received high-dose immunosuppressive therapy; ANF was detected in seven patients (Figure 1).

From left to right—antibodies to antigens of cardiomyocyte nuclei (antinuclear antibodies), endothelium, cardiomyocytes, smooth muscle, and fibres of the conduction system. On the ordinate axis—multiplicity of antibody titre increase in relation to the threshold value of 1:40. The normal range is marked with pink.

**Figure 2:**
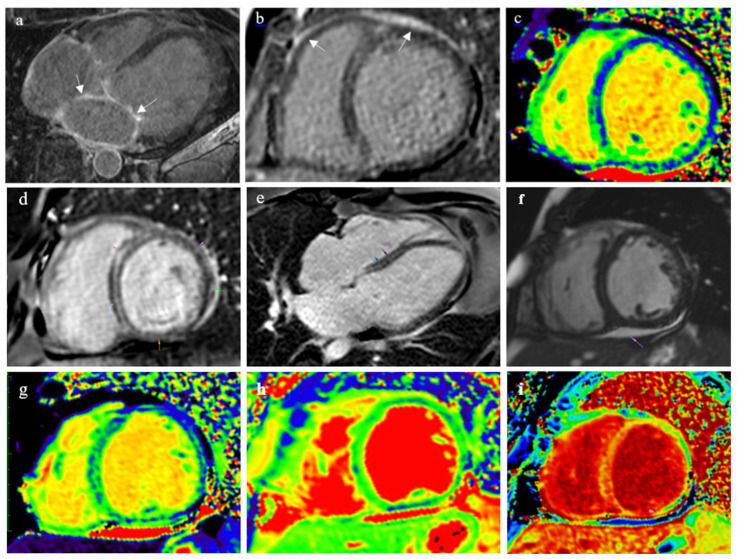
Cardiac MRI in patients with cardiomyopathy and post-COVID myocarditis. Patient A, 44-year-old man results (**a**–**c**): late gadolinium enhancement in atria ((**a**)—4-chamber view) and ventricles subepicardially ((**b**)—short axis view), T1 map ((**c**)—short axis view, diffuse T1 changes along the posterior septal segment). Patient with laminopathy, 44-year-old man, results (**d**–**i**): intramyocardial late gadolinium enhancement throughout the entire interventricular septum at basal and mid-levels, subepicardial in posterior and anterolateral segments ((**d**)—short axis view, (**e**)—4-chamber view); (**f**)—short axis view, pericardial effusion due to myopericarditis; (**g**)—short axis view, native T1 map, increase in T1 relaxation time to a maximum of 1107 ms in the posterior septal segment (N 995 ± 32), in the anterior septal, anterolateral and posterior segments; (**h**)—short axis view, T2 map, increase in T2 relaxation time to a maximum of 56 ms in the posterior septal segment (N 46 ± 3), in the anterior septal and posterolateral segments; (**i**)—short axis view, ECV map (plotted using CMR CVI 42 software, GE Healthcare), an increase in the extracellular volume (ECV) fraction maximally along the interventricular septum to 38 ± 3%.

All patients underwent cardiac MRI (Figure 2). It was very difficult to interpret the results in the context of CMP. Nevertheless, all patients showed subepicardial late gadolinium enhancement (LGE) typical of myocarditis and no signs of oedema were observed. Other Lake Louise criteria included pericardial effusion, increased ECV and signs of myocardial hyperaemia in various combinations.

**Figure 3:**
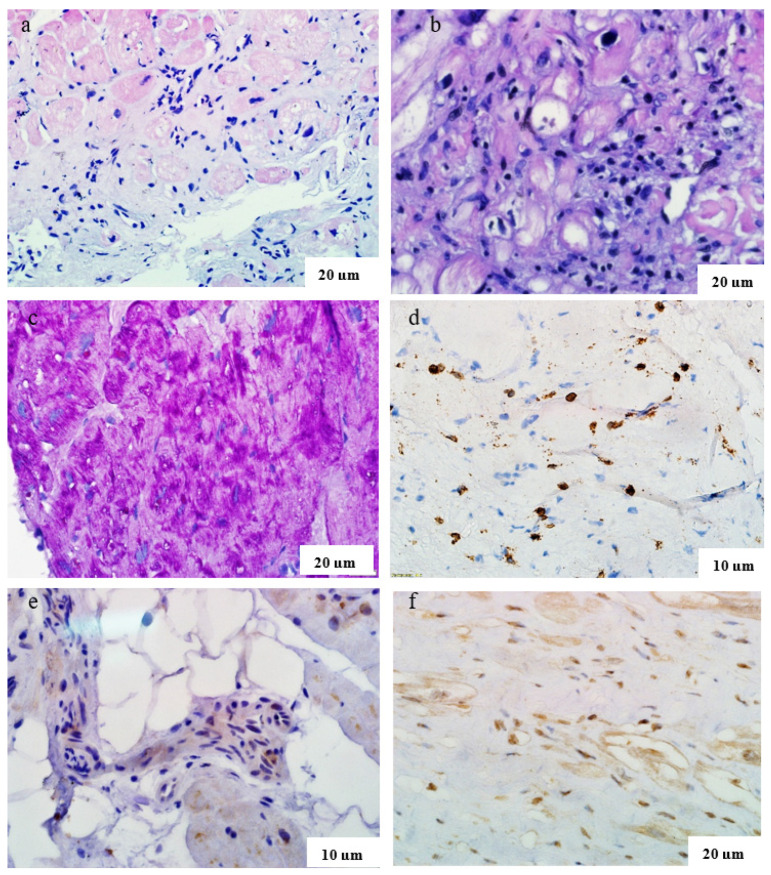
Results of morphological study of myocardium in patients with post-COVID myocarditis and various cardiomyopathies. Haematoxylin and eosin staining ((**a**,**b**)—lymphohistiocytic infiltration in myocardium, necrosis of several cardiomyocytes), PAS reaction ((**c**), accumulation of PAS-positive substance in cardiomyocytes in a patient with Danon’s disease), IHC study with antibodies to CD3-lymphocytes ((**d**), more than 7 cells in 1 mm^2^), to Spike protein (**e**) and nucleocapsid (**f**) of SARS-CoV-2 with a positive reaction in infiltrate cells, endothelium and several cardiomyocytes.

EMB results showed active lymphocytic myocarditis in all six patients (Figure 3, Table 1). Coronavirus RNA was detected by PCR in three patients, and the presence of coronavirus proteins in the myocardium was confirmed by IHC in all patients. In one case (a 59-year-old man with LVNC), parvovirus B19 DNA was also detected in the myocardium.

Let us characterise some of these patients in more detail.

**Patient A, 44-year-old man**, was admitted with complaints of dyspnoea on minimal exertion and at rest, episodes of dizziness associated with low blood pressure, heaviness in the right subcostal region, increase in abdominal size, general weakness.

Suffered from arterial hypertension for more than 15 years, diagnosed with diabetes mellitus in 2017, not treated. Paroxysmal atrial fibrillation was diagnosed at the age of 25, with PVCs of up to 20,000 per day. On EchoCG, the EF was 48% (2017). In 2019, radiofrequency pulmonary vein isolation was performed with effect; there were no pathological findings at coronarography. May 2020: COVID-19 in severe form. Within two months, dyspnoea and abdominal size increased. In November, EchoCG showed dilatation of all heart chambers, EF 21%, systolic pulmonary artery pressure (SPAP) 60 mmHg.

On admission, the skin was swarthy; swelling of the shins and feet was observed; no rales during lung auscultation; BP 110/60 mm Hg, HR 80 beats per minute, heart tones were rhythmic, muffled, no murmurs; abdomen was enlarged due to ascites, the liver protruded from under the edge of the rib arch by 4–6 cm. Blood tests showed Hb 179 g/L (haematocrit 57.7%), no general inflammatory changes, ferritin 549.4 mcg/L, signs of moderate cholestasis, hyperglycaemia up to 21.4 mmol/L. With an aim to evaluate the immunologic activity of myocarditis, the titre of blood anticardiac antibodies (Ab) was assessed: Ab to antigens (Ag) of cardiomyocyte nuclei—1:80 (N—abs); Ab to endothelial Ag—1:80 (N ≤ 1:40) (N ≤ 1:40); Ab to cardiomyocytes Ag—1:80 (N ≤ 1:40); Ab to smooth muscle Ag—1:320 (N ≤ 1:40); Ab to cardiac conductive fibres Ag—1:160 (N ≤ 1:40).

ECG revealed low voltages of QRS complexes and decreased amplitude of R waves in chest leads. In HM 9159, PVCs of two morphologies and non-sustained ventricular tachycardia were registered. EchoCG: left ventricular end-diastolic diameter (LVED) 6 cm (2.91 cm/m^2^), end-diastolic volume (EDV) 194 mL (94.17 mL/m^2^), end-systolic volume (ESV) 154 mL (74.76 mL/m^2^), LVEF 21%, VTI 7.5 cm, dp/dt 909 mmHg, no areas of asynergy. Restrictive diastolic dysfunction was observed (E/A 2.6), SPAP 52 mmHg. There was no evidence of pulmonary embolism.

MRI showed signs of iron overload in the posterior wall of the LV, prolonged T1 relaxation time of “native” myocardium in all LV segments at the medium level, subepicardial LGE in the posterior wall, intramyocardial LGE in the septal segments of the LV (volume of fibrotic changes 11%) and in the atrial walls (Figure 2). A heterozygous mutation in the HFE gene was revealed, ARVC DNA-diagnosis is in progress. EMB revealed large fibro-fatty fields, active lymphocytic myocarditis with endotheliosis phenomena, Perls reaction demonstrated single iron granules in cardiomyocytes. Coronavirus RNA was identified in the myocardium (10 months after COVID-19). The patient’s condition was considered as a combination of post-COVID myocarditis and CMP (haemochromatosis with local cardiac involvement, possible ARVC). Along with cardiotropic, anticoagulant and glucose-lowering therapy, treatment with methylprednisolone 16 mg/day was started, and two bloodletting procedures were performed with normalisation of the ferritin level.

The patient’s condition improved and 8 months later (October 2021) blood tests were within normal limits, there were no rhythm disturbances in HM (on the background of amiodarone administration), in EchoCG—LV EDD 5.2 cm, EDV 116 mL, ESV 49 mL, EF 61%, VTI 14.5 cm, and SPAP 28 mm Hg. Such a prominent positive dynamic allows us to speak about the leading role of post-COVID myocarditis in severe decompensation, but it does not exclude primary CMP in any way.

**Patient K, 44-year-old woman**, was admitted on 8 September 2021 with complaints of palpitations, weakness on moderate exertion, dyspnoea on walking, frequent nonproductive cough and weight gain.

Family history is burdened: patient’s father died suddenly at the age of 60 y.o.; her first son was diagnosed with HCM and Wolf–Parkinson–White phenomenon, pathogenic mutation was revealed on the 7th exon of the *LAMP2* gene (chrX:119576459_119576489del;). He died at the age of 16 y.o. due to progressive heart failure. The second son (15 years old) is healthy.

Since school age, the patient suffered from palpitations. Since 2017, she had moderate dyspnoea, EF was 54%, frequent supraventricular extrasystoles were recorded on ECG. Electrophysiological study in 2018 showed no data for additional conduction pathway, tachycardia was not induced. In 2018, EchoCG showed LV hypertrophy up to 17 mm and the condition was considered as HCM. In 2019, ECG showed atrial flutter and AF. In August 2020, radiofrequency ablation (RFA) was performed without full effect. In October 2020, she had COVID-19, after which the patient noticed an increase in dyspnoea and palpitations. In June 2021, during hospitalisation for CHF decompensation, she had bilateral hydrothorax, EF 45%, frequent PVCs and non-sustained VT. Coronary angiography did not reveal any abnormal findings, and an EMB was performed to rule out storage disease and/or myocarditis.

On admission, physical examination revealed obesity (BMI 35.6 kg/m^2^) and oedema of the shins; no rales on lung auscultation. Heart sounds were rhythmic, muffled, and single PVCs were heard; HR 105 d/min, BP 110/80 mmHg. The liver and spleen were not enlarged. In blood tests—hypochromic anaemia (Hb 114 g/l), C-reactive protein 8 mg/l, Ab to antigens (Ag) of cardiomyocyte nuclei—1:40 (N—abs); Ab to cardiac conductive fibres Ag—1:160 (N ≤ 1:40).

ECG showed frequent premature supraventricular contractions, no evidence of LV hypertrophy, flattened T waves. HM recorded up to 14,000 supraventricular extrasystoles and 3000 polytopic PVCs. EchoCG demonstrated LV hypertrophy up to 12 mm, EDD 5.3 cm, EDV 124 mL, ESV 84 mL, EF 34–41%, VTI 9.5 cm, dp/dt 909 mm Hg, LV diastolic dysfunction of restrictive type (E/A 3.4, Dt 95 ms, Emed 3.8 cm/s, E/Emed 28, Elat 8.9–12 cm/s, E/E 20), both atria 85 mL, SPAP 41 mmHg.

Myocardial biopsy specimens (Figure 3a,c) revealed definite active myocarditis; typical small-vessel lesions in the form of endotheliitis were consistent with a coronavirus aetiology; no SARS-Cov-2 RNA or cardiotropic virus DNA was detected by PCR (one year after COVID-19). According to the results of DNA diagnostics, a diagnosis of Danon’s disease was confirmed: the same pathogenic mutation in a heterozygous state was detected in the *LAMP2* gene as in her son.

Cardiotropic and anti-inflammatory therapy was prescribed (methylprednisolone 24 mg/day with gradual reduction to 2 mg/day, azathioprine 100 mg/day). Dyspnoea decreased and palpitations became significantly less frequent. On EchoCG 8 months later, the LVEF was 49% and no signs of pulmonary hypertension were observed. However, taking into account the nature of her primary CMP, complete recovery is not expected, as well as the effect of repeated RFA.

**Patient S, a 59-year-old man**, was admitted to the clinic on 8 November 2021 with complaints of dyspnoea on slow walking over minimal distances, transient swelling of the shins and feet, and palpitations accompanied by a feeling of retrosternal compression.

The patient’s father died of stroke at the age of 48 and his mother of heart failure at the age of 65. Throughout his life, the patient tolerated physical activity well and worked as a cook. There were no abnormalities on ECG or EchoCG about 3 years ago. In December 2020, he had COVID-19 and treatment was provided at home. From June 2021, dyspnoea and palpitations appeared, and at the end of the summer, leg oedema, jaundice and epigastric pain began. ECG revealed atrial flutter, EchoCG showed dilatation of all ventricles, EF was 15–19%. Coronarography demonstrated intact coronary arteries. Sinus rhythm was successfully restored, cardiotropic therapy was started without clear effect and heart transplantation was recommended.

On admission to the clinic, the patient had swelling of the shins. HR 56/min, BP 110 and 70 mmHg. In blood tests there was a tendency to leukopenia (4.1 thousand x109/l), specific ANP 1:320, antibodies to antigens of cardiomyocytes and smooth muscle 1:160. The ECG showed sinus bradycardia and signs of LV hypertrophy. There were no rhythm disturbances in HM. On EchoCG LV EDD 7.0 cm, EDV188 mL, ESV144 mL, EF 23%, VTI 8.8 cm, dp/dt 568 mmHg, severe restrictive dysfunction (E/A 4,4) and pulmonary hypertension SPAP 51 mmHg, and the presence of a non-compaction layer of the LV myocardium was first noted. MRI confirmed LVNC, with subendocardial (up to 20%) LGE in the inferior septal region.

EMB (Figure 3e,f) showed endocardial sclerosis with adjacent thrombotic masses, active lymphocytic myocarditis with thrombovasculitis, coronavirus proteins were detected in infiltrate cells and cardiomyocytes (11 months after COVID-19). No cardiotropic virus DNA was detected. The onset of myocarditis led to decompensation of previously asymptomatic familial LVNC. Methylprednisolone 24 mg/day was prescribed, and cardiotropic and anticoagulant therapy was continued.

The patient’s condition gradually improved, and at the 5-month echocardiogram LVEF was 40%, VTI 12.5 cm, with a significant reduction in left ventricular dimensions (LV EDD 6.1 cm, EDV 163 mL, ESV 96 mL, LA 59 mL). LVNC was preserved. Therapy was continued.

**Patient K, 39-year-old man**, was admitted to the clinic on 9 March 2023 with complaints of dyspnoea on moderate physical exertion. The patient’s mother had atrial fibrillation and dyspnoea at the age of 40, and a pacemaker was implanted due to bradysystole; at the age of 57, she developed angina pectoris with sharp escalation of dyspnoea and an increased troponin level, but her coronary arteries were intact, and infarction-like myocarditis was diagnosed. Due to the ineffectiveness of complex therapy, the patient’s mother underwent a successful heart transplantation at the age of 58.

The patient tolerated considerable physical exertion (professional dancing), but at the age of 30, HM revealed paroxysms of atrial fibrillation and he stopped exercising and began to notice moderate dyspnoea. In July 2021 he was vaccinated against a new coronavirus infection with “Sputnik V”, and in December 2021 he suffered from COVID-19 (PCR +) in a mild form. Since February 2022, he noticed increasing dyspnoea, which occurred in November with minimal exertion and at night, and in December he experienced a single episode of syncope. In January 2023 he consulted a cardiologist. ECG showed QS complexes in leads V1-V4 and atrial fibrillation and EchoCG showed a significantly reduced EF (30%). Cardiac MRI showed subepicardial LGE of the anterior and inferior LV myocardium. Treatment with sacubitril-valsartan, torasemide, spironolactone, apixaban and dapagliflozin was started. On the background of amiodarone administration, restoration of sinus rhythm with a tendency to bradycardia was observed and the drug was discontinued.

On admission to the clinic, there were no significant signs of CHF, ECG showed atrial fibrillation with HR 70/min, anterior hemiblock, QS complexes in leads V1-V2, insufficient rise in amplitude of the R wave in leads II-III, aVF, V1-V3, during HM normosystole was registered without rate control therapy, there were no ventricular rhythm disturbances. EchoCG showed an unenlarged left ventricle (LVR 5.1 cm, LVR 146 mL, CSR 75 mL) with moderately reduced contractility (EF 48%, VTI 13 cm), no asynergy zones. Antibody titres to cardiomyocyte nuclei antigens (1:80) and to intercalated disc antigens (1:160) were moderately elevated. The condition was considered to be a combination of primary cardiomyopathy and post-COVID myocarditis in the resolution phase. Hydroxychloroquine was added to the treatment and the patient’s condition remains stable.

Whole exome sequencing revealed heterozygous VUCs in the MYH7 (exon 16, p. Thr619Ile) and MyBPC3 (exon 2, p. Ala31) genes. A search for similar mutations in the patient’s parents is underway.

**Figure 4:**
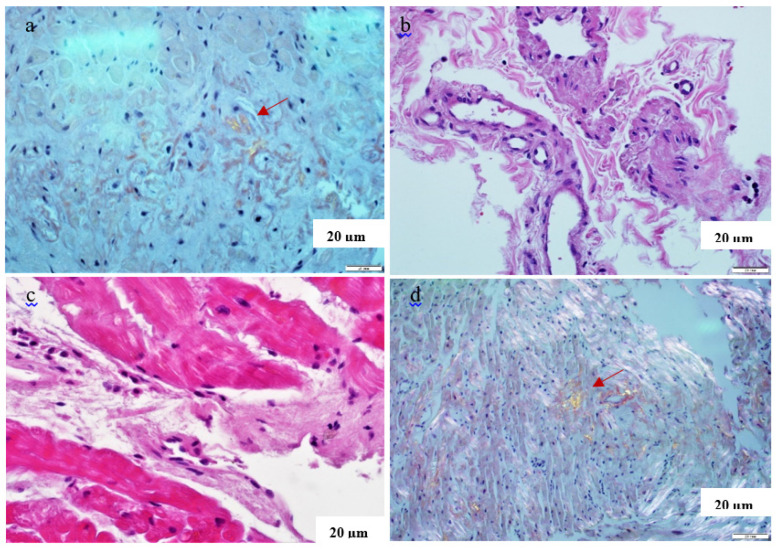
Endomyocardial biopsy specimens of the right ventricle in patients with a combination of AL amyloidosis and post-COVID myocarditis. Congo red with evaluation in polarising light ((**a**,**d**), Congo-positive masses in cardiomyocytes and vessel walls, apple-green luminescence of amyloid shown by arrows) and haematoxylin and eosin staining ((**b**,**c**), perivascular and interstitial lymphohistiocytic infiltration). Explanation in the text.

Finally, we present three cases of patients with a combination of **newly diagnosed AL amyloidosis and post-COVID myocarditis**. In two of them, EMB of the RV was performed after COVID-19 and not only confirmed the diagnosis of amyloidosis, but also revealed signs of coronavirus myocarditis with detection of RNA and viral proteins in the myocardium, Figure 4. Clinically, superimposed myocarditis manifested as an increase in heart failure symptoms, ventricular arrhythmias or anginal pain with unchanged coronary arteries. Given that all AL amyloidosis treatment regimens include corticosteroids, no specific treatment for myocarditis was prescribed.

A third patient with AL amyloidosis of the heart presented with acute decompensation after COVID-19, with EF declining from 54% to 21% within one month and anasarca development. EMB was not performed. Chemotherapy (dexamethasone, bortezomib, cyclophosphamide and daratumumab) combined with diuretics was administered, but no clinical improvement was observed. There was no increase in anti-cardiac antibodies and MRI showed diffuse, predominantly subendocardial. Empirical steroid therapy was continued and EF increased to 34%. Chemotherapy for amyloidosis was also restarted.

**Treatment**. Four out of ten patients with post-COVID myocarditis and CMP were treated with methylprednisolone monotherapy at a mean starting dose of 24 [16; 24] mg/day with subsequent reduction to a maintenance dose of 4–6 mg/day. Patients with AL-amyloidosis were treated with chemotherapy including steroids. One patient was prescribed hydroxychloroquine. Two patients were not prescribed immunosuppressive therapy (due to pulmonary infection and lack of EMB data).

Patients also received standard cardiotropic therapy, taking into account the characteristics of the underlying disease: ACE inhibitors or sartans (30%), ARNIs (50%), *β*-blockers (60%), mineralocorticoid receptor antagonists (80%), dapagliflozin (60%), amiodarone (60%), loop diuretics (90%), and anticoagulants (80%).

**Outcomes**. Two deaths from end-stage heart failure with development of asystole were recorded in patients with AL amyloidosis. The remaining patients showed positive clinical dynamics and a significant increase in left ventricular ejection fraction (see Table 1).

## 4. Discussion

There are practically no descriptions of post-COVID myocarditis and primary CMP in the literature. Let us mention the only case of possible acute myocarditis in a previously asymptomatic patient with LVNC: acute decompensation with elevated troponin levels and a decline in EF up to 20% that developed during COVID-19, and EchoCG revealed definite LVNC [9]. Such a scenario of LVNC diagnosis is one of the most typical because of the slow progression of LV systolic dysfunction. Post-COVID myocarditis “revealed” LVNC in two patients we observed, including a patient with acute decompensation one month after delivery.

There is evidence of a high incidence of cardiac damage in pregnant women with COVID-19: in one study it was 10%, the disease was severe (mean EF 38%, hospitalisation in intensive care in 100% of cases, need for mechanical ventilation in 86.6%), and postpartum mortality was 13.3% [10]. The majority of patients appear to have acute myocarditis, but the development of true peripartum CMP cannot be excluded, nor can the manifestation of primary CMP, as in our case.

Danon’s disease presented in our cohort, especially in women, is one of the phenocopies of HCM and its combination with post-COVID myocarditis is unique. Only one case of pre-COVID myocarditis in Danon’s disease has been reported in the literature [11]. Repeated administration of immunosuppressive therapy did not have a complete effect, which is related to the initial genetic myocardial pathology. In our case, the patient tolerated two pregnancies well, she had no significant systolic dysfunction for a long time, and only the addition of post-COVID myocarditis led to a pronounced decompensation at the age of 44 years (while her son died at the age of 16 years). This is due to the peculiarities of the phenotypic manifestations of X-linked diseases.

AL-amyloidosis is not a classical hereditary CMP and develops as a result of plasma cell dyscrasia, but the character of the cardiac involvement corresponds to HCM with signs of restriction and a gradual decrease of LV contractility. The group of H.P. Schultheiss at the Charité showed morphological signs of myocarditis on EMB in 48% of 56 patients with AL-amyloidosis and its negative influence on prognosis was proven [12]. In our cases, EMB confirmed not only myocarditis but also coronavirus persistence in the myocardium, which is unlikely to alter the chemotherapy regimen but requires careful monitoring. Monitoring blood levels of anti-cardiac antibodies is also useful.

Finally, we should mention the possible role of coronavirus vaccination in the induction and exacerbation of myocarditis in patients with primary CMP. At least two of our patients developed symptoms of myocarditis after vaccination both before and after COVID-19. A case of probable postvaccination myocarditis has been described in a patient with a left ventricular form of ARVC with a mutation in the DSP gene (predisposing to myocarditis) and a history of two episodes of probable myocarditis [4]. In our opinion, vaccination should be avoided in CMP patients with post-COVID myocarditis (as well as with a history of myocarditis of other aetiology).

## 5. Conclusions

The pandemic period confirmed the concept that genetically defective myocardium is a favourable background for the development of chronic myocarditis, including viral myocarditis. Myocarditis is one of the most important transient (epigenomic) factors in the manifestation and progression of primary CMP. Post-COVID myocarditis has been diagnosed in patients with various primary CMPs (LVNC, dilated restrictive CMP, Danon’s disease, laminopathy, and AL amyloidosis) within 4 to 11 months after COVID-19. Myocarditis was manifested by the onset/increase of arrhythmias and heart failure. In most cases, myocarditis required morphological confirmation due to difficulties in the differential diagnosis and immunosuppressive therapy decisions. Both coronavirus persistence in the myocardium (in five out of six patients) and autoimmune mechanisms played a role in the development of post-COVID myocarditis. Corticosteroids proved to be quite effective in the treatment of myocarditis and the issue of cytostatic prescription needs to be studied further.

## Figures and Tables

**Table 1:** Basic clinical characteristics of patients with cardiomyopathies and post-COVID myocarditis.

Sex	m	f	f	m	m	m	m	f	m	m
Age	44	44	55	59	36	52	67	31	57	39
Clinical diagnosis	ARVC, hemochromatosis	Danone	RCM	LVNC	DCM	AL-amyloidosis	AL-amyloidosis	LVNC	AL-amyloidosis	DCM
Genetic findings	*HFA*	*LAMP2 p.E298* Afs*38	*MyBPc3* p.Gln969*		*LMNA p.Lys78Asn*					*MYH7 p.Thr619Ile (III)*
CMP family history (+—family burden)	+	+	+	+	+					+
Anticardiac antibodies (+ numbers proportional to elevation level)	+++	+++	+++	++	++	+++	++	++	++	++
LF EF, % initial/ follow-up	21/55	41/49	63/60	23/43	33/37	20/? N/A	42/N/A	32/44	25/35	30/48
MRI (+ numbers proportional to myocarditis signs intensity)	++	++	++	++	+++	+	+	++	++	++
Biopsy	LM	LM	LM	LM	N/A	LM	LM	N/A	N/A	N/A
SARS-CoV-2 RNA in myocardium (+—positive; –—negative	+	–	+	+	N/A	–	–	N/A	N/A	N/A
SARS-CoV-2 proteins, IHC, myocardium (+—positive; –—negative	+	+	+	+	N/A	+	+	N/A	N/A	N/A
Steroids	+	+	–	+	–	–	+	+	+	–
Death	–	–	–	–	–	+	–	–	+	–

CMP—cardiomyopathy; LM—lymphocytic myocarditis; IHC—immunohistochemistry; N/A—not available.

## Data Availability

The data presented in this study are available on request from the corresponding author.
